# Reduced chondroitin sulfate content prevents diabetic neuropathy through transforming growth factor-β signaling suppression

**DOI:** 10.1016/j.isci.2024.109528

**Published:** 2024-03-18

**Authors:** Hajime Ishiguro, Takashi Ushiki, Atsuko Honda, Yasuhiro Yoshimatsu, Riuko Ohashi, Shujiro Okuda, Asami Kawasaki, Kaori Cho, Suguru Tamura, Tatsuya Suwabe, Takayuki Katagiri, Yiwei Ling, Atsuhiko Iijima, Tadahisa Mikami, Hiroshi Kitagawa, Akiyoshi Uemura, Kazunori Sango, Masayoshi Masuko, Michihiro Igarashi, Hirohito Sone

**Affiliations:** 1 Departments of Hematology, Endocrinology, and Metabolism, Graduate School of Medical and Dental Sciences, Niigata university, Niigata, Japan; 2 Department of Neurochemistry and Molecular Cell Biology, Graduate School of Medical and Dental Sciences, Niigata University, Niigata, Japan; 3 Division of Pharmacology, Graduate School of Medical and Dental Sciences, Niigata University, Niigata, Japan; 4 Divisions of Molecular and Diagnostic Pathology, Graduate School of Medical and Dental Sciences, Niigata University, Niigata, Japan; 5 Division of Bioinformatics, Graduate School of Medical and Dental Sciences, Niigata, Japan; 6 Center for Research Promotion, Graduate School of Medical and Dental Sciences, Niigata University, Niigata, Japan; 7 Division of Hematology and Oncology, Graduate School of Health Sciences, Niigata University, Niigata, Japan; 8 Departments of Transfusion Medicine, Cell Therapy and Regenerative Medicine, Medical and Dental Hospital, Niigata University, Niigata, Japan; 9 Hematopoietic Cell Transplantation Niigata University Medical and Dental Hospital, , Niigata University, Niigata, Japan; 10 Neurophysiology & Biomedical Engineering Lab, Interdisciplinary Program of Biomedical Engineering, Assistive Technology and Art and Sports Sciences, Faculty of Engineering, Niigata University Niigata, Niigata, Japan; 11 Laboratory of Biochemistry, Kobe Pharmaceutical University, Kobe, Japan; 12 Department of Retinal Vascular Biology, Nagoya City University Graduate School of Medical Sciences, Nagoya, Japan; 13 Diabetic Neuropathy Project, Department of Diseases and Infection, Tokyo Metropolitan Institute of Medical Science, Tokyo, Japan

**Keywords:** Molecular biology, Neuroscience, Immunology, Omics, Transcriptomics

## Abstract

Diabetic neuropathy (DN) is a major complication of *diabetes mellitus*. Chondroitin sulfate (CS) is one of the most important extracellular matrix components and is known to interact with various diffusible factors; however, its role in DN pathology has not been examined. Therefore, we generated CSGalNAc-T1 knockout (T1KO) mice, in which CS levels were reduced. We demonstrated that diabetic T1KO mice were much more resistant to DN than diabetic wild-type (WT) mice. We also found that interactions between pericytes and vascular endothelial cells were more stable in T1KO mice. Among the RNA-seq results, we focused on the transforming growth factor β signaling pathway and found that the phosphorylation of Smad2/3 was less upregulated in T1KO mice than in WT mice under hyperglycemic conditions. Taken together, a reduction in CS level attenuates DN progression, indicating that CS is an important factor in DN pathogenesis.

## Highlights

• CSGalNAcT1-KO did not suffer from diabetic neuropathy following induced hyperglycemia
• Retinal pericytes in CSGalNAcT1-KO were resistant to anti-PDGFβR
• WT diabetic neuropathy involved TGF-β signaling, whereas CSGalNAcT1-KO did not

## Graphical abstract

**Figure:**
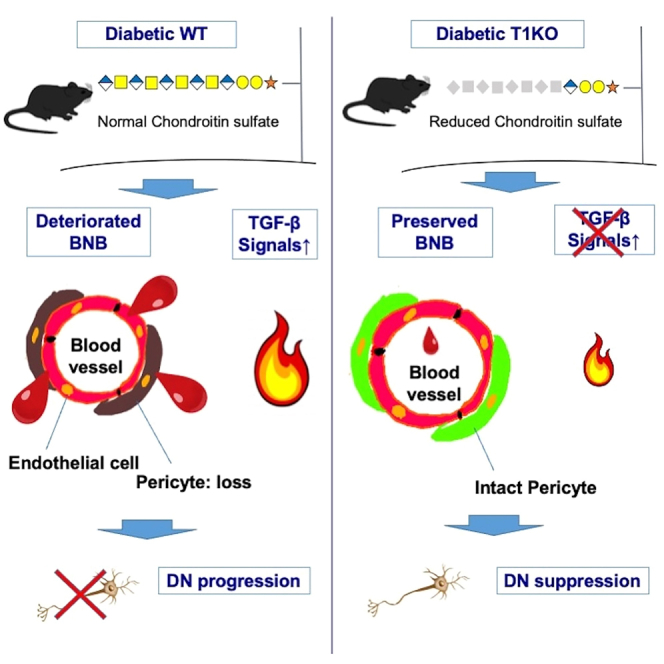

## Introduction

*Diabetes mellitus* (DM), defined simply on the basis of hyperglycemia, is the most common metabolic disorder,^1^^,^^2^ and chronic hyperglycemia induces many complications such as retinopathy and nephropathy. Among the complications, diabetic neuropathy (DN) is a distal polyneuropathy characterized by the major die-back style of neurodegeneration that damages peripheral neurons.^3^^,^^4^^,^^5^^,^^6^ DN is the most common complication through the prediabetic and diabetic stages, and substantially affects patients by causing pain, numbness, and autonomic dysfunction, as well as reducing the patients’ quality of life.^3^ How hyperglycemia leads to peripheral neurodegeneration has not been clearly elucidated compared to other complications.^7^^,^^8^ Several intracellular signaling pathways, such as those for ER stress, oxidative damage, apoptotic mechanisms, or glycolytic side pathways, have been suggested to be involved in the pathogenesis of DN. However, the underlying causative mechanisms have not been firmly established.^5^ As a result, essentially no effective drugs have been identified for DN therapy or prevention, with the only current preventive and therapeutic option being the maintenance of optimal blood glucose control.^9^

DN is associated with inflammation resulting from abnormal inter-cellular communication associated with microangiopathies.^10^ This suggests that the mechanism of DN involves not only intracellular, but also extracellular signals related to cell-cell communications or interactions. Thus, we focused on one of the major extracellular matrix components, chondroitin sulfate (CS), which is thought to be a potential modulator of intracellular and intercellular events.^11^ CS is a glycosaminoglycan that is composed of long repeated disaccharide units of [*N*-acetyl-D-galactosamine (GalNAc)-D-glucuronic acid (GlcA)] with esterified sulfate residues.^11^ CS, present in large amounts in various tissues, including those of the nervous system,^12^ has a highly negative charge because of its sulfate residues; as a result, CS is known to interact with various diffusible factors such as growth factors, cytokines, or chemokines, acting as a receptor or as a trap.^13^ Thus, CS is one of the key components in inflammatory signaling, and combines with a core protein to form CS proteoglycan (CSPG).

To analyze the physiological and pathological roles of CS, we established mice with knockout (KO) of CSGalNAcT1 (T1),^13^ an enzyme that catalyzes the first step of CS-specific synthesis pathways and is considered to be rate-limiting for CS synthesis.^14^^,^^15^ CS is known as a potent and abundant inhibitor of axon growth and regeneration after injury, however, compared to wild-type (WT), T1KO has shown considerably accelerated recovery from spinal cord injury,^16^ as well as milder symptoms of experimental encephalitis (a model of human multiple sclerosis).^17^ One of the characteristics of DN in humans is that the axon regeneration of damaged peripheral nerves is impaired;^18^ thus, these results may indicate the possibility of milder DN or resistance to developing DN in T1KO with hyperglycemia. In addition, another factor in diabetes complications such as nephropathy has been suggested to be the relationships of the extracellular matrix, including CS.^19^

Here, we used T1KO and examined whether the onset of DN was altered in this mouse model. Under conditions of hyperglycemia induced by streptozotocin (STZ; a type 1 diabetes model), which causes hypoalgesia and sciatic nerve degeneration in wild-type (WT) mice,^5^ T1KO mice seldom exhibit such symptoms and nerve degeneration. However, T1KO mice did not exhibit abnormal values for biochemical indices related to DN, and the peripheral nerves were largely intact. These results suggest that the peripheral nerves of T1KO mice are resistant to hyperglycemic stimuli. In addition, the developmental stability of pericytes in the presence of anti-PDGFRβ antibody treatment was much improved in T1KO compared to WT mice. However, the reason for the observed resistance to DN was not fully understood by analyzing neuronal metabolism and intracellular responses; thus, RNA-seq analysis was performed and revealed the presence of abnormal transforming growth factor (TGF)-β signaling^20^ in diabetic WT mice, which was abrogated in diabetic T1KO mice. Taken together, it was determined that nerves in T1KO mice are resistant to STZ-induced hyperglycemia.

## Results

### Chondroitin sulfate content in the sciatic nerve was reduced in CSGalNAc-T1 knockout mice compared with wild type

**Figure 1:**
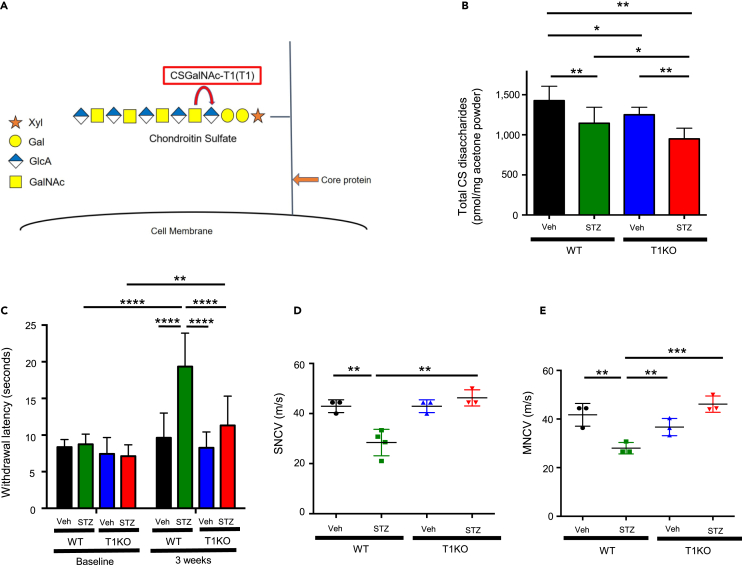
CS was reduced in sciatic nerves and DN progression evaluated by electrophysiological analyses was prevented in the diabetic state of T1KO mice (A) Schematic diagram of GAG synthesis. The steps in CS synthesis are as follows: (1) synthesis of the tetrasaccharide linker that is attached to core proteins, starting from the Xylt1 activity; (2) attachment of an *N*-acetylgalactosamine (GalNAc) to the linker; (3) addition of glucuronic acid (GlcA) to the GalNAc and subsequent polymerization of the disaccharide backbone (GalNAc-GlcA); and (4) sulfation at several sites of these glycosyl derivatives. In CS synthesis, T1 transfers GalNAc to the linker. (B) The amount of CS in the sciatic nerves, after three weeks during hyperglycemia. Mean ± SD is shown with *∗*p < 0. 05 and *∗∗*p < 0. 01 for comparison, one-way *ANOVA* with Tukey’s multiple comparisons test, n = 10 mice per group. (C) Comparison of paw withdrawal latency in the radiant heat test at baseline (pre-treatment) and after three weeks of hyperglycemia. Mean ± SD is shown with *∗∗*p < 0.01, *∗∗∗*p < 0.001 and *∗∗∗∗*p < 0.0001 for comparison, two-way *ANOVA* with Bonferroni’s multiple comparison test, n = 9 in the non-diabetic WT group, n = 12 in the non-diabetic T1KO group, n = 12 in the diabetic WT group, and n = 11 in the diabetic T1KO group. (D) Sensory nerve conduction velocity (SNCV) of sciatic nerves. Mean ± SD is shown with ∗∗p < 0.01 for comparison, one-way ANOVA with Tukey’s multiple comparisons test, n = 3 each in the non-diabetic WT group, diabetic WT group, and diabetic T1KO group, and n = 4 in the non-diabetic T1KO group. (E) Motor nerve conduction velocity (MNCV) of sciatic nerves. Mean ± SD is shown with *∗∗*p < 0.01 and *∗∗∗*p < 0.001 for comparison, one-way ANOVA with Tukey’s multiple comparisons test, n = 3 per group.

T1 is the most important enzyme for the regulation of CS synthesis (Figure 1A; see Introduction). T1 gene knock-out resulted in a 20–30% reduction in total CS disaccharides in the sciatic nerve compared to WT mice (Figure 1B). T1KO did not affect blood glucose levels or STZ-induced diabetes (Table 1). Specifically, there were no significant differences in blood glucose levels between the non-diabetic WT group and the non-diabetic T1KO group. In addition, blood glucose levels were significantly higher in the respective STZ groups than the vehicle groups three days after STZ injection. No significant differences in body weight between WT and T1KO mice under diabetic conditions were observed, although mice of both genotypes showed reduced weights compared to their counterparts in their respective non-STZ groups (Table 1).

**Table 1:** Body weight and blood glucose levels in mice

	WT-vehicle	WT-STZ	T1KO-vehicle	T1KO-STZ
BW(g) at baseline	18.9 ± 1.1	19.1 ± 1.0	17.6 ± 1.5	18.1 ± 1.7
BW(g) at 3 weeks	21.6 ± 1.1	∗17.2 ± 2.0	19.7 ± 2.4	∗∗17.4 ± 2.6
Blood glucose (mg/dL)	170 ± 32	∗425 ± 74	162 ± 37	∗∗450 ± 64

Body weight (BW) at baseline and 3 weeks after injection, and blood glucose level at day 3 following streptozotocin (STZ) injection in all groups. Vehicle groups received citrate buffer alone. Mean ± Standard deviation (SD) is shown. There are no significant differences in baseline BW. ∗p < 0.0001 for comparison of wild type (WT)-vehicle with WT-STZ. ∗∗p < 0.0001 for comparison of CSGalNAc-T1 knockout (T1KO)-vehicle with T1KO-STZ. There were no significant differences in blood glucose between vehicle-treated WT mice and T1KO mice, as well as between STZ-treated diabetic wild-type mice and diabetic T1KO mice, n = 9–12 per group.

### Diabetic neuropathy progression was attenuated in diabetic CSGalNAc-T1 knockout neurons, while glucose metabolism was unchanged

The heat radiant test was performed to evaluate peripheral nerve dysfunction. This test is able to measure and evaluate the degree of analgia using the thermal stimulation of the hind paw. There were no significant differences in thermal stimulation response times before STZ or vehicle injection; however, responses in the diabetic WT mice were significantly prolonged three weeks after injection. In contrast, the response time in diabetic T1KO mice was significantly shorter than in diabetic WT mice (Figure 1C). Motor nerve conduction velocity (MNCV) and sensory nerve conduction velocity (SNCV) in the sciatic nerve, the gold standard parameters for evaluating DN, were also decreased in diabetic WT mice; however, these velocities were preserved in diabetic T1KO mice (Figures 1D and 1E). From these results, we suspected that T1KO attenuated DN-dependent analgia and produced a neuroprotective effect.

**Figure 2:**
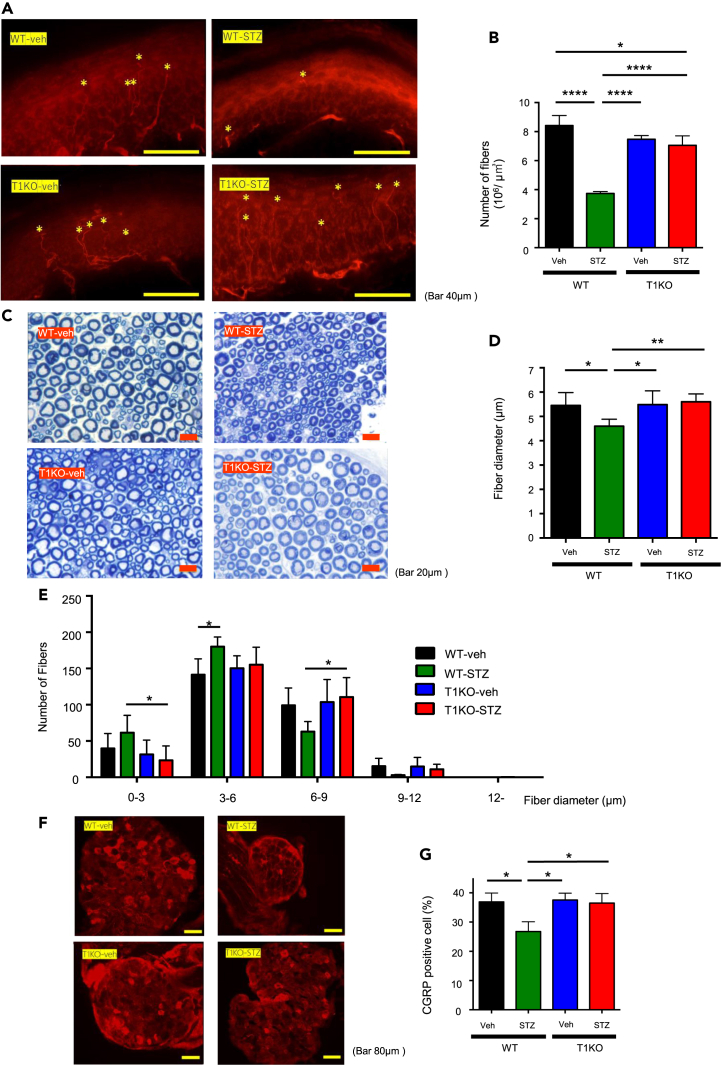
DN progression was attenuated in diabetic T1KO, as evaluated by morphological and immunohistological analyses (A) Representative fluorescence images of nerve fibers in footpads after three weeks of hyperglycemia. *Red,* Tuj-1. ∗, intraepidermal nerve fiber. (B) Intraepidermal nerve fiber density in footpads of mice after three weeks of hyperglycemia. Mean ± SD is shown with *∗*p < 0.05 and *∗∗∗∗*p < 0.0001 for comparison, one-way ANOVA with Tukey’s multiple comparisons test, n = 3 each in the non-diabetic WT group, diabetic WT group and diabetic T1KO group, and n = 4 in the non-diabetic T1KO group. (C) Representative micrographs of toluidine blue-stained sciatic nerve cross-sections in each group. (D) Average fiber diameter of sciatic nerves. The average of 300 blue-stained fibers per mouse was calculated after three weeks of hyperglycemia. Mean ± SD is shown with ∗p < 0.05 and ∗∗p < 0.01 for comparison, one-way ANOVA with Tukey’s multiple comparisons test, n = 5 each in the non-diabetic WT group and diabetic WT group, n = 6 each in the non-diabetic T1KO group and diabetic T1KO group. (E) Histogram of nerve fiber diameters of sciatic nerves. Horizontal sections were taken at 5 mm distal to the neuromuscular junction and 300 blue-stained nerve fibers were counted. Mean ± SD is shown. *∗*p < 0.05 for comparison, one-way ANOVA with Tukey’s multiple comparisons test, n = 5 each in the non-diabetic WT group and diabetic WT group, n = 6 each in the non-diabetic T1KO group and diabetic T1KO group. (F) Representative imaging of CGRP staining in the DRG. (G) The proportion of CGRP-positive neurons in the DRG. The neurons were excised from three DRGs per mouse and calculated. Mean ± SD is shown with *∗*p < 0.05 for comparison, one-way ANOVA with Tukey’s multiple comparisons test, n = 3 per group.

Pathological characterization of peripheral nerve damage was performed by evaluating intraepidermal nerve fiber density (IENFD) in the footpad using quantitative analysis of neuronal class III β-tubulin (Tuj-1) staining. Regarding plantar IENFD, diabetic WT mice showed a significantly reduced the density of epidermal fibers in the plantar footpads. Conversely, the IENFD of diabetic T1KO mice was preserved (Figures 2A and 2B). Toluidine blue staining of sciatic nerves was conducted to investigate whether there were any morphological changes caused by DN in more proximal nerves. Although the mean myelinated fiber diameter was significantly smaller in diabetic WT mice than in non-diabetic WT mice, the fiber diameter in diabetic T1KO mice was preserved (Figures 2C and 2D).

The distribution of individual fibers is shown in Figure 2E, indicating that the fiber diameter in the diabetic WT group was smaller as a whole than in the other groups (Figure 2E). In contrast, analysis of the G/T ratio, representing the myelination of nerves, indicated that there were no significant differences among the groups (Figure S1A). These results suggest that diabetic T1KO, in which CS amounts were reduced, showed neuroprotective effects against DN.

Initially, we considered whether such changes in DN might be caused by differences in glucose uptake in dorsal root ganglia (DRG) neurons; however, metabolomic analysis revealed that specific metabolic pathways, such as those of glycolysis or the TCA cycle, were not found to be substantially changed (Figure S2).

### Peptidergic neurons were protected and apoptosis-initiating genes were suppressed in the dorsal root ganglia of diabetic CSGalNAc-T1 knockout

To investigate which types of peripheral nervous system (PNS) neurons were preserved, we examined the levels of calcitonin gene-related peptide (CGRP) and Substance P in hyperglycemic DRG neurons, since both CGRP and Substance P are considered to be important neurotransmitters for thermoception.^21^^,^^22^ Immunohistochemistry revealed a loss of CGRP-positive neurons in diabetic WT DRG, which contains a cluster of sensory neuronal cell bodies. In contrast, DRG neurons were protected in diabetic T1KO mice (Figures 2F and 2G). Moreover, there were no significant differences in substance P-positive neuron ratios (Figures S1B and S1C).

**Figure 3:**
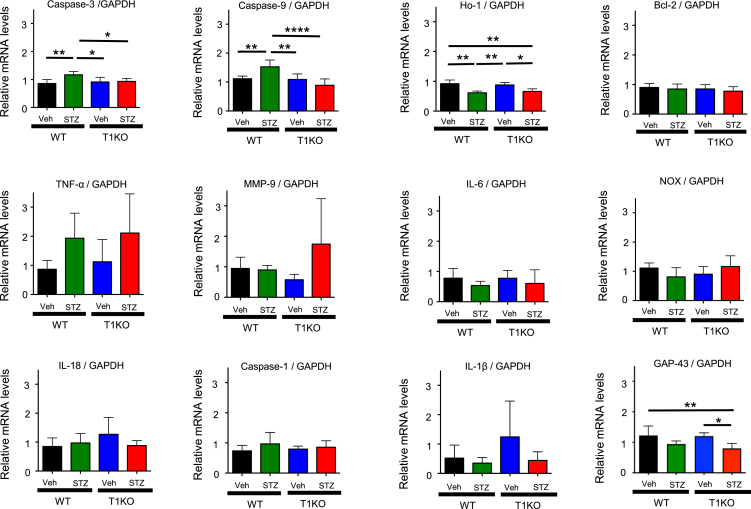
Genes related to neuronal cell death or nerve degeneration were not elevated in diabetic T1KO mice mRNA expression levels were evaluated in DRG extracted at 3 weeks. Mean ± SD is shown with *∗*p < 0.05, *∗∗*p < 0.01 and *∗∗∗∗*p < 0.0001 for comparison, one-way *ANOVA* with Tukey’s multiple comparisons test, n = 6 per group in Caspase-3 and Caspase-9, n = 4 per group in Ho-1, Bcl2, TNF-α, MMP-9, NOX and GAP-43, n = 3 per group in IL-6, IL-18, Caspase-1 and IL-1β.

We then analyzed the gene expression patterns in DRG by real-time PCR and confirmed the elevation of the well-known apoptosis-initiating genes caspase-3 and caspase-9 in diabetic WT mice. In comparison, T1KO mice did not exhibit an induction in these genes. However, no significant differences were detected between WT and T1KO mice in the diabetic state (Figure 3) for several genes previously reported in DN pathogenesis, including Bcl-2 (anti-apoptosis gene), inflammation markers (TNF-α, MMP-9, and IL-6), reactive oxygen-related enzymes (Ho-1 and NOX), and inflammasome members (IL-18, IL-1β, and Caspase 1). The marker of axon growth or regeneration GAP-43 was not upregulated in diabetic T1KO mice (Figure 3), suggesting that the preservation of PNS neurons is not due to axon regeneration after inflammatory degenerative responses.

### Blood-nerve barrier integrity was maintained in diabetic CSGalNAc-T1 knockout

In the context of the above results, T1KO probably plays an important role in suppressing DN. However, previously reported mechanisms involved in DN, affecting endogenous DRG pathways in particular, were not significantly related to the CS-dependent inhibition of DN development. Thus, we focused on interactions among neurons and other cells. The blood-nerve barrier (BNB) is the functional structure that is thought to restrict molecular transport to PNS neurons,^23^ similar to the blood-brain barrier in the central nervous system. Vascular cells, including endothelial cells and pericytes, are components of the BNB. Pericytes are known to express NG2 (CSPG4) on their surface;^24^ thus, we hypothesized that the nature of the pericytes of T1KO mice may change and become resistant to DN.

**Figure 4:**
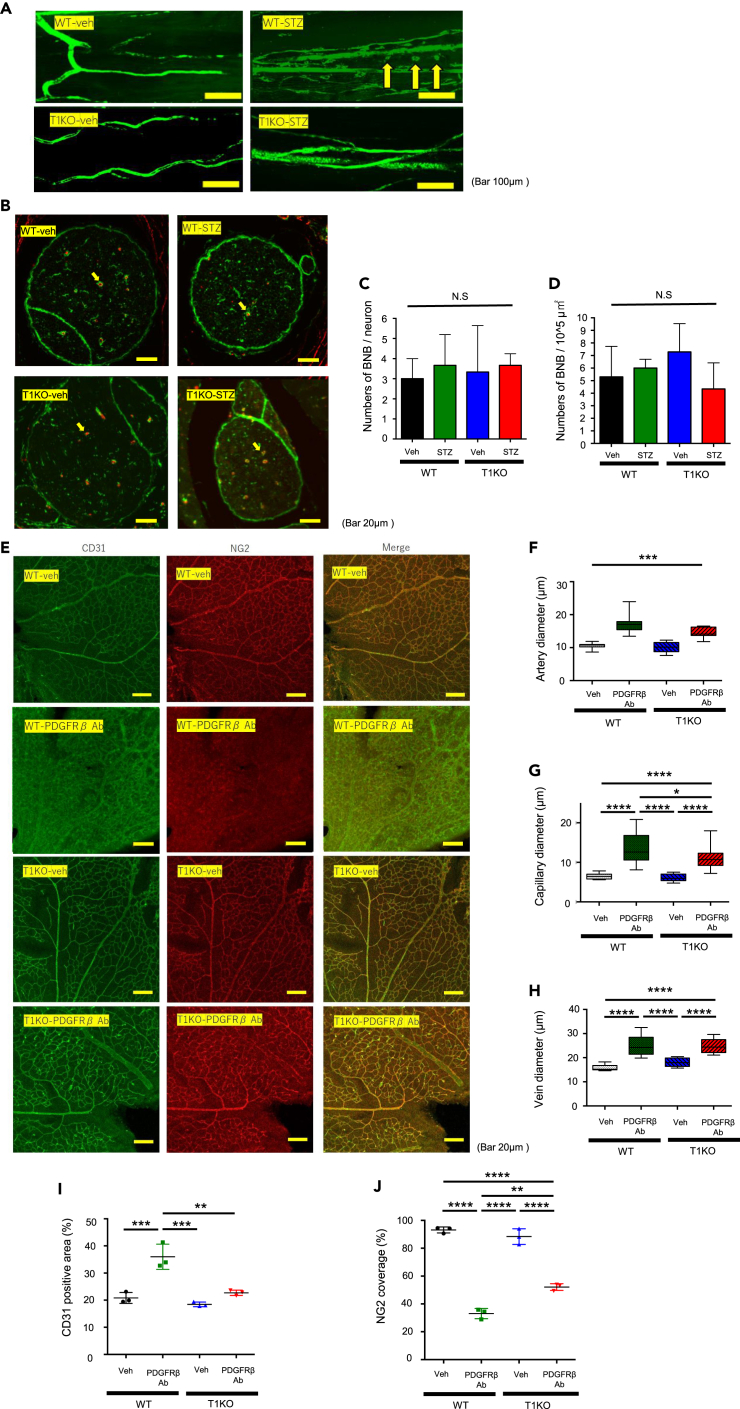
BNB integrity against hazardous stimuli was maintained in T1KO mice (A) Representative images of sciatic nerves injected with 70 kDa FITC-dextran in the absence and presence of hyperglycemic stimuli. *Arrows* indicate extra-vascular leakage of FITC-dextran. Note that the WT-STZ group, but not the T1KO-STZ group, showed leakage from the vessels. (B) Representative IHC images of sciatic nerve cross-sections. BNB was defined as a structure in which PDGFRβ (a pericyte marker)-positive cells surround CD31 (an endothelial cell marker)-positive cells. *Red*: CD31; *Green*: PDGFRβ. A*rrows*; BNB structures. The number of BNBs per sciatic nerve (C) and (D) per area at three weeks after STZ injection. Mean ± SD is shown, n = 3 per group. (E) Representative IHC images of retinal vessels on postnatal day 8 (P8) after anti-PDGFRβ Ab injection. *Green*: CD31; *Red*: NG2. (F–H) Quantification of retinal vessel diameters after anti-PDGFRβ Ab administration, compared to the control groups; (F) arteries, (G) capillaries, and (H) veins. (I and J) Measurement of CD31-positive areas (I) and NG2 coverage (J) in P8 retina after anti-PDGFRβ Ab administration, compared to the control groups. Mean ± SD is shown with *∗*p < 0.05, *∗∗*p < 0.01, *∗∗∗*p < 0.001, and *∗∗∗∗*p < 0.0001 for comparison, one-way ANOVA with Tukey’s multiple comparisons test, n = 3 per group.

BNB collapse was reported to be an important factor in DN.^23^ We proposed that BNB collapse was the most probable candidate mechanism; therefore, we further characterized the phenotype of diabetic T1KO by measuring the permeability of the endoneurial endothelium in sciatic nerves. The endoneurial capillaries of diabetic WT mice three weeks after STZ injection were permeable to 70 kDa FITC-dextran; based on the observation that FITC dyes leaked out from the blood vessels. In contrast, those of diabetic T1KO mice were not leaky, and the dyes remained within the vessels (Figure 4A).

To observe the structural changes in more detail, the BNB was defined as the structure in which PDGFRβ (a pericyte marker) surrounds CD31 (an endothelial cell marker) by immunohistochemistry (IHC),^23^ and these structures were counted. The results indicated that there were no significant differences in BNB number (Figures 4B–4D). Therefore, these data suggested that the BNB numbers themselves were maintained in the diabetic state of both WT and T1KO, although BNB function was impaired in WT and maintained in T1KO.

### Anti-PDGFRβ antibody injection collapsed the structure of retinal blood vessels in wild type but not in CSGalNAc-T1 knockout

Based on the above results, we suspected that the characteristics of pericytes in T1KO were altered to be resistant to diabetic complications. To evaluate this in detail, we examined pericyte characteristics in the retinal vessels of T1KO mice, as it is much easier to observe pericyte function in the retina than in peripheral nerves.

We previously reported that the injection of anti-PDGFRβ mAb (clone APB5) into the mouse eye on postnatal day 1 (P1) induced structural collapse of retinal vessels, i.e., the enlargement of retinal vessels and reduced pericyte coverage.^25^ Injection of anti-PDGFRβ Ab (Figure 4E) on P1 resulted in wider diameters of retinal arteries (Figure 4F) and capillaries (Figure 4G) in WT mice as compared to T1KO mice. As a result, CD31-positive areas were increased on P8 (Figure 4I).

Staining with anti-NG2 Ab, a marker of pericytes, revealed that NG2 coverage of blood vessels was decreased to a greater extent and led to retinal pericyte depletion following the injection of anti-PDGFRβ Ab in WT compared to T1KO mice (Figure 4J). There were no significant differences in vain diameter (Figure 4H). Taken together, these results indicated that pericytes in T1KO mice were more resistant to PDGFRβ antibody than those in WT mice, suggesting that the vascular system in T1KO mice is more resistant to certain external stresses, such as hyperglycemia, compared to that in WT mice.

### CSGalNAc-T1 knockout mice exhibit reduced activation of transforming growth factor-β-related signaling under diabetic conditions

T1KO was characterized as being less susceptible to noxious stimuli (heat); however, the relationship to CS reduction (Figure 1) and the expression of specific molecules remains to be identified (Figure 3). To detect key molecules mediating the specialized properties of T1KO, we performed a comprehensive RNA-seq analysis of DRGs three weeks after the confirmation of the initiation of diabetic symptoms. Although various changes, such as in genes associated with the nervous system, between individual groups (Figure S3) were observed, we focused on patterns related to the phenotype of the radiant heat test. Specifically, only the diabetic WT group exhibited increased values compared to the other three groups, and genes with similar tendencies accounted for about 10% of TGF-β signaling-related genes. These results indicated that TGF-β signaling pathways were candidate regulators of DN (Table S1).^26^ The expression levels of TGF-β receptor 1 (*Tgfbr1*) and other TGF-β superfamily related genes were dramatically altered; namely, bone morphogenetic protein receptor type 1A (*Bmpr1a*), activin receptor type 1B (*Acvr1b*), growth differentiation factor 1 (*Gdf1*), which encodes a secreted ligand of the TGF-β superfamily, *Smad5,* which is involved in the TGF-β superfamily signaling pathway, and rho associated coiled-coil containing protein kinase 1 (*Rock1*), which is a downstream pathway of TGF-β signaling. In addition, a similar tendency was observed for *Bmpr1a*^23^ (Figure S4A) as well as xylosyltransferase 1(*Xylt1)*, the initiation enzyme of CS synthesis (Figure S4B), catalyzing the linkage between xylose and a serine residue in the core proteins^11^ (see also Figure 1A). Thus, we proposed that TGF-β signaling is a key factor that can explain the anti-DN effects of T1KO.

**Figure 5:**
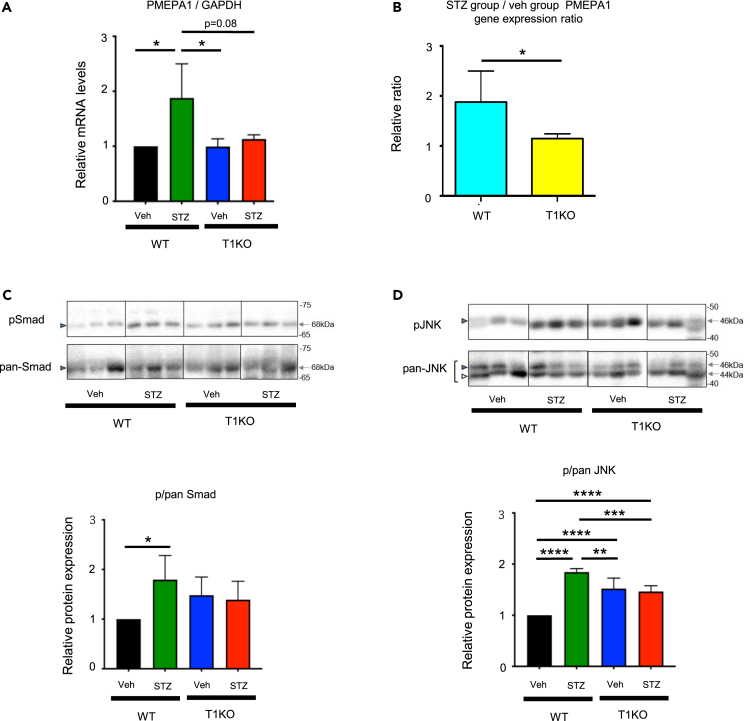
Neuroprotective effects of T1KO against diabetic stimuli may involve attenuated TGF-β signaling in diabetic T1KO (A) PMEPA1 gene expression in MS-1 cells. DRG neurons derived from WT and T1KO mice were cultured and their culture supernatant was used to stimulate MS-1 cells. (B) PMEPA1 gene expression in STZ-treated (relative to vehicle) WT and T1KO mice. (C) Representative images of Smad2/3 and pSmad2 proteins detected by western blotting using phospho-specific Abs against their linker domains (29, 30) in sciatic nerves and the pSmad/Smad ratios in sciatic nerves. (D) Representative images of JNK and pJNK proteins detected by western blotting in the sciatic nerves and the pJNK/JNK ratios in sciatic nerves. Mean ± SD is shown with *∗*p < 0.05, *∗∗*p < 0.01, *∗∗∗*p < 0.001, and *∗∗∗∗*p < 0.0001 for comparison, one-way *ANOVA* with Tukey’s multiple comparisons test, n = 6 per group.

To investigate the role of TGF-β in T1KO mice, we examined TGF-β signaling after hyperglycemic stimuli. The gene expression of prostate transmembrane protein, androgen-induced 1 (PMEPA1) in MS-1 cells was evaluated. The PMEPA1 level was significantly higher when MS-1 cells were immersed in the culture supernatant of diabetic WT neurons, compared to that of the non-diabetic WT neurons. However, there was no significant difference in PMEPA1 expression between MS-1 cells immersed in the culture supernatants of non-diabetic and diabetic T1KO mice (Figure 5A).

The PMEPA1 gene expression ratio of the diabetic/non-diabetic groups was significantly higher in WT than in T1KO (Figure 5B). TGF-β binds to TGF-β receptor II, which induces the activation of TGF-β receptor I and phosphorylation of Smad2/3 in the cytoplasm.^23^ In addition, it is reported that the phosphorylation of a specific site in the linker region of Smad2 by JNK, a mitogen-activated protein kinase (MAPK) family member, stimulates Xylt1 gene expression.^27^ Thus, we examined the phosphorylation of Smad2 in WT and T1KO. The results revealed that both pSmad/Smad and pJNK/JNK ratios were elevated in diabetic WT compared to non-diabetic WT mice; however, the ratios were unchanged between diabetic and non-diabetic T1KO mice in the sciatic nerve (Figures 5C and 5D).

## Discussion

Our present results involving hyperglycemic models in T1KO mice are highly novel. Specifically, conditions that produced DN in WT animals did not result in the development of DN-dependent symptoms in T1KO animals, including hypoalgesia or nerve conduction delay, and pathological indicators of nerve degeneration. Although there are a number of metabolic stress pathways proposed to be involved in the pathogenesis of DN that are dependent on hyperglycemia, these neuronal abnormalities have not been sufficiently investigated to explain the mechanisms involved in DN (Figure 3). Herein, we used a novel view of DN pathogenesis to examine the possibility that the extracellular matrix component CS modulates intracellular molecular communications.

Nerve damage in diabetic WT mice showed the activation of apoptotic signals (Figure 3); however, these signals were not activated in diabetic T1KO mice. This indicated that T1KO results in reduced susceptibility to STZ-inducible apoptosis. Although oxidative stress has been reported to be related to this complication,^5^^,^^28^ contradictory results were reported concerning HO-1 levels. In the present study, diabetic WT mice showed decreased HO-1, whereas HO-1 levels were not significantly decreased in diabetic T1KO mice, suggesting that T1KO does not obviously affect oxidative stress regulation (Figure 3).

### Prevention of nerve degeneration, or nerve regeneration?

If nerves that should be damaged are otherwise preserved, we must consider two possibilities: one is the occurrence of the prevention of nerve degeneration, and the other possibility is that nerve regeneration is occurring after damage. While it is difficult to determine which mechanism is correct, the former possibility is considered the likely explanation of the present observations. The sciatic nerves in diabetic T1KO mice were preserved intact, and the nerves were not considered to have been regenerated since the molecular marker of axon extension and regeneration, GAP-43 (Figure 3),^29^^,^^30^ was not elevated, and RNA-seq did not show elevated levels of axon guidance molecules (Figure S3). These results indicate that hyperglycemia did not damage the sciatic nerve in T1KO mice, with the reduced CS levels preventing hyperglycemic toxicity in the sciatic nerve.

### Pericyte stability in CSGalNAc-T1 knockout

Pericytes have recently been demonstrated to perform important roles in barrier function, such as in the blood-brain barrier^31^ and blood-nerve barrier,^23^ and are involved in human diseases through microangiopathy.^32^^,^^33^ The present results suggest that the prevention of diabetic neuropathy in T1KO mice is dependent on cellular interactions, such as pericyte function. We examined the loss of pericytes or their reduced coverage of vessels in the PNS of mice; however, we were unable to quantify such changes. It is thought that our mouse model did not present these pathological changes because the duration of hyperglycemia in our model is much shorter than in human patients with diabetes who present with pathological changes. Thus, we attempted to demonstrate changes in pericytes between WT and T1KO mice in the retina, which are involved in blood-retina barrier formation^25^ (Figure 4). WT retinal pericytes were highly susceptible to the anti-PDGFRβ antibody, as previously reported,^25^ while those of T1KO were largely resistant to this treatment (Figure 4). These results suggest that PDGFRβ-dependent retinal barrier function, i.e., PDGF signaling involved in the formation of the BRB via PDGFRβ, is related to the amount of CS, and reduced CS inhibits anti-PDGFRβ antibody toxicity. Additional experimental data are needed to fully clarify the relationships between the present retinal results and the hyperglycemic PNS results.

### Transforming growth factor-β signaling may be involved in streptozotocin -induced diabetic neuropathy

We examined intracellular changes in metabolic pathways in diabetic T1KO; however, we did not identify specific pathways, since all of the abnormal pathways in diabetic WT were not altered in diabetic T1KO, indicating that T1KO sciatic nerves were quite resistant to hyperglycemia.

RNA-seq revealed a tendency for the elevation of several TGF-β group receptors in the diabetic WT group (Figure S4A), and we successfully detected the elevation of linker domain phosphorylation in diabetic WT mice (Figures 5C and 5D). Thus, we concluded that the neuronal damage in the diabetic WT group involved TGF-β signaling. In addition, the expression of the TGF-β target gene TMEPA1^34^ was elevated in diabetic WT mice, indicating that TGF-β signaling was activated and TMEPA1 up-regulation was induced via TGF-β signaling in diabetic WT mice but not T1KO mice (Figure 5A).

We also found that among WT mice, the phosphorylation of the Smad2/3 linker domain and JNK was elevated in the diabetic group; however, the phosphorylation of these molecules was not elevated in T1KO mice (Figures 5C and 5D). TGF-β signaling is known to be involved in other diabetic complications, such as diabetic nephropathy and retinopathy^35^^,^^36^; thus, it is likely that this signaling pathway is a pathogenic factor of DN. Moreover, it was previously suggested that TGF-β signals regulate Xylt1 expression via Smad phosphorylation, thereby inducing CS synthesis.^37^^,^^38^

Xylt1 is the first step enzyme in CS linker domain synthesis.^15^ We found that Xylt1 showed a tendency for elevation in the diabetic WT group compared to the other three groups (Figure S4B). Taken together, it appears that in the course of neuronal damage, diabetic WT mice exhibit elevations in TGF-β signaling, Smad2/3 phosphorylation, Xylt1 expression, and ultimately, the synthesis of CS. In other words, hyperglycemic diabetic neuropathy in WT mice may be caused by CS synthesis, and CS may be an exacerbating factor of DN in WT mice. In contrast, it is suggested that T1KO mice exhibit insufficient TGF-β-related signaling even under diabetic conditions, and CS synthesis was reduced in the sciatic nerve, thereby suppressing DN progression in diabetic T1KO mice.

According to previous reports, TGF-β signaling was shown to up-regulate the enzymes involved in CS synthesis.^39^^,^^40^ In those reports, increased CS production in the extracellular matrix induced by TGF-β, i.e., an inflammatory mediator, was thought to be related to fibrosis or scar formation resulting from the effects of TGF-β. Although we did not demonstrate a direct relationship between CS and DN, it is likely that CS upregulation may promote TGF-β signaling and exacerbate extracellular matrix remodeling to promote an increased inflammatory response.

### Summary of findings obtained in this study

Our article pursues many different potential molecular/cellular mechanisms related to DN. In conclusion, mechanisms in diabetic T1KO mice prevent the elevation of apoptosis-initiating genes, BNB dysfunction, and TGF-β-related signaling activation, and therefore, prevent DN progression.

Extracellular CS may be involved in mediating diabetic complications related to barrier function in the blood-nerve^25^ or blood-retinal barriers,^25^ and inhibition of CS synthesis may represent a novel drug target for DN therapeutics. In addition, the pathway shown here could help to explain the lower rates of DN in Asians,^41^ although no genetic evidence of defects in CS synthesis in patients with DN has been obtained so far.

### Limitations of the study

In the present study, the impact of CS on DN conditions was assessed in mice, but has not been examined in humans. It is also unclear whether T1KO mice exhibit alterations in interactions between the remaining CS and various diffusible factors, such as growth factors, cytokines, or chemokines. Lastly, the detailed biochemical mechanisms related to hyperglycemia and activated TGF-β signaling remain to be fully elucidated at the cellular level.

## STAR★Methods

### Key resources table

**Table:** 

REAGENT or RESOURCE	SOURCE	IDENTIFIER
**Antibodies**		

β-tubulin (Mouse anti-Neuron-specific β-Ⅲ tubulin antibody)	R&D SYSTEM	BAM1195
CGRP (Mouse Monoclonal anti-CGRP antibody)	SIGMA-Aldrich	C7113
substance P (Rat Monoclonal anti-substance P antibody)	Abcam	ab7340
CD31 (Rabbit Monoclonal anti-CD31 antibody) (for sciatic nerve)	Abcam	ab56299
PDGFRβ (Rabbit Monoclonal anti- PDGFRβ antibody).	Abcam	ab32570
CD31 (Purified Rat Monoclonal anti-CD31 antibody) (for retina)	BD Pharmingen	557355
NG2 (Rabbit Polyclonal anti-NG2 antibody)	Merck Millipore	AB5320
goat anti-mouse polyclonal IgG Alexa Fluor 555 (for β-tubulin and CGRP)	Abcam	ab150114
goat anti-rat polyclonal IgG Alexa Fluor 555 (for substance P)	Abcam	ab150158
goat polyclonal anti-rat Alexa Fluor 488 (for retina CD31)	Abcam	ab150157
goat polyclonal anti-rabbit Alexa Fluor 568 (for retina NG2)	Abcam	ab175471
Anti-Smad2/3 antibody	R&D Systems	AF 3797
anti-phosphorylated Smad2 (pSmad S245/250/255) antibody	Cell Signaling Technology	#3104
anti-SAPK/JNK antibody	Cell Signaling Technology	#9252
anti-phosphorylated SAPK/JNK (Thr183/Tyr185)(81E11) antibody	Cell Signaling Technology	#4668
Recombinant anti-mouse PDGFRβ mAb(clone APB5)	Uemura et al.^22^	N/A

**Chemicals, peptides, and recombinant proteins**		

Streptozotocin	Fujifilm Wako Pure Chemical	195-15154

**Deposited data**		

RNA sequences data	Takara Bio, Inc	https://doi.org/10.17632/fsj9c8mfbn.2

**Experimental models: Organisms/strains**		

CS-GalNAc T1 KO mice / C57BL/6	Watanabe et al.^14^	N/A

**Oligonucleotides**		

Primers for Taqman Assay	Thermo Fisher Scientific	See Table S2
Primers for PMEPA1 (MS-1 cells RT-PCR) Fwd: 5’-TGG AGT TCG TGC AAA TCG TG-3’ Rev: 5’- GCT GTG TCG GCT GAT GAA G -3’	Intergated DNA Technologies	N/A
Primers for 1GAPDH (MS-1 cells RT-PCR) Fwd: 5’- ATG TGT CCG TCG TGG ATC TGA-3’ Rev: 5’- TTG AAG TCG CAG GAG ACA ACC T-3’	Thermo Fisher Scientific	N/A
TaqMan Fast Advanced Master Mix	Thermo Fisher Scientific	Cat#4444557
THUNDERBIRD SYBR qPCR mix	TOYOBO	QPS-201
SuperScript 3 Reverse Transcriptase	Thermo Fisher Scientific	Cat#18080093
RNaseOUTRecombinantRibonucleaseInhibitor	Thermo Fisher Scientific	Cat#10777019
dNTP Mix	Thermo Fisher Scientific	Cat#18427013
Oligo(dT)	Thermo Fisher Scientific	Cat#18418020

**Software and algorithms**		

GraphPad Prism v.6	Prism-graphpad.com	https://www.graphpad.com/scientific-software/prism/

### Resource availability

#### Lead contact

Further information and requests for resources and reagents should be directed to and will be fulfilled by the lead contact: Prof. Michihiro Igarashi, MD/PhD; tarokaja@med.niigata-u.ac.jp.

#### Materials availability

This study did not generate new unique reagents.

#### Data and code availability

• All original code is available in this paper’s supplemental information.
• Code availability: RNA sequencing data have been deposited at the DDBJ Sequence Read Archive (http://trace.ddbj.nig.ac.jp/dra/) under accession number PSUB021567 (see https://data.mendeley.com/datasets/fsj9c8mfbn/2).
• Any additional information required to reanalyze the data reported in this work paper is available from the lead contact upon request.

### Experimental model and study participant details

#### Animals and ethics statement

T1KO mice were described previously^16^ and were maintained on the C57BL/6N background.^16^ Six-week-old male mice were randomly assigned to experiments for 3 weeks. All animal experiments in this study were approved by the Animal Ethics Committees of Niigata University (approval number: SA01310).

### Method details

#### Experimental diabetes induction

STZ (WAKO, Tokyo, Japan) was injected into 6-week-old male mice for five days (50 mg/kg/day via intraperitoneal injection). Blood glucose was measured by OneTouch® UltraVue (Johnson and Johnson, New Brunswick, NJ, USA) three days after the STZ injection, and was defined as true diabetes, not prediabetes, if the serum glucose levels were higher than 250 mg/dl.

#### Quantification of glycosaminoglycans

Analysis of CS in sciatic nerves was performed using enzymatic treatments and HPLC-based quantification.^14^^,^^42^

#### Thermal nociceptive response

The heat radiant test was performed before and 3 weeks after STZ injection. In brief, the mice were placed on a thermal stimulation meter (7371-plantar test, Ugo Basile SRL, Veneto, Italy), followed by measurement of the time until their licking of the hind paw or moving from the heated plate. The test was repeated three times per mouse at least 10 min apart. The mean of three measurements was taken as the latency to paw withdrawal.

#### Immunohistochemistry

Epidermal footpads were obtained from the plantar hind foot, and DRGs were collected from the lumbar spine (L3-5) and stained.^43^ The footpads and DRGs were stained using anti β-tubulin antibody (1:500 dilution, R&D Systems, Minneapolis, MN 55413, USA), anti-calcitonin gene related peptide (CGRP) (1:500 dilution, SIGMA-Aldrich, St. Louis, MO, USA) or substance P (1:500 dilution, Abcam, Cambridge, UK (England)) antibodies. To quantify intraepidermal nerve fiber density (IENFD), the labeled nerve fibers in the epidermis were counted under a fluorescence microscope, and the fiber density (number of nerve fibers x 10^6^ μm^3^) was calculated. To quantify CGRP and Substance P positive cells in DRGs, sections were observed under a fluorescence microscope. The fluorescence intensity was shown to be bimodal; of the bimodal peaks, the cell groups belonging to the higher peak were defined as CGRP or Substance P-positive, and we counted the ratio of positive cell numbers. Three DRGs per mouse underwent quantitative analysis, and a mean value per mouse was calculated. All micrographs were taken using a BZ-9000 microscope (Keyence, Osaka, Japan). For IHC of sciatic nerves, the tissues were fixed in 10% formalin and embedded in paraffin, and 3-μm-sections were cut and then stained. Briefly, the sections were deparaffinized, rehydrated and autoclaved at 120°C for 20 min in Histofine antigen retrieval buffer [pH 9.0] (Nichirei Bioscience, Tokyo, Japan) and then the primary antibody, PDGFRβ (1:50 dilution, Abcam), was reacted using Tyramide SuperBoost Kits with Alexa Flour 568 (ThermoFisher Scientific, Waltham, MA, USA) according to the manufacturer's instructions. Thereafter, the sections were autoclaved as previously described at pH9 and another primary antibody, CD31 (1:50 dilution, Abcam), was reacted using Tyramide SuperBoost Kits with Alexa Flour 488 (ThermoFisher Scientific). IHC of whole-mount retinas was processed as described previously.^44^ Anti-CD31 (1:500 dilution, BD Bioscience, Franklin Lakes, NJ, USA) and anti-NG2 (1:1000 dilution, Merck Millipore, Darmstadt, Germany) were used as primary antibodies. Images of sciatic nerves and retinas were acquired with an inverted confocal microscope (FV1200/IX83, Olympus, Tokyo, Japan). Images were analyzed using NIH ImageJ (version 1.52, http://iimagej.nih.gov/ij/).

#### Motor and sensory nerve conduction velocity measurements

Motor and sensory nerve conduction velocities (MNCV and SNCV, respectively) were measured under isoflurane anesthesia. MNCV and SNCV in sciatic nerves were assessed by analysis of evoked muscle action potentials from the gastrocnemius muscle, and of the hind limb, respectively. Needle electrodes (NM-030T, Nihon Koden Ltd., Tokyo, Japan) were used for stimulation and recording of electromyograms. The stimulation sites were the distal and proximal points from the recording sites with a 10 mm gap between the stimulus sites. Square wave stimulation with a constant voltage of 10 V and a duration of 0.1 ms was generated by the stimulator (SEN-2201, Nihon Koden Ltd.). The electromyogram was recorded with LabChart ver7 (ADInstruments, Colorado Springs CO, USA) via pre and main amplifiers (DPA-2004PN, DPA2004N, Dia Medical System Co., Tokyo, Japan; Powerlab/8sp, ADInstruments, respectively). Nerve conduction velocity was calculated by dividing the distance between the stimulating sites by the differences of each electromyogram’s latency.

#### Vascular permeability assay

For endoneurial capillary permeability assessment, 300 μl of 70 kDa FITC-dextran (25 mg/ml, Sigma-Aldrich) was injected into each mouse. After 30 min, the mice were sacrificed and FITC-dextran extravasation was evaluated using an inverted confocal microscope (FV1200/IX83, Olympus) from whole mount sciatic nerves.

##### Staining of myelin sheaths

Small tissue samples of the resected sciatic nerve were embedded in epoxy resin (Epon 812).^45^ Epon-embedded specimens were cut into 700 nm semi-thin transverse sections and stained with 1% toluidine blue. Myelin was stained blue in ring shapes, while the axons within the ring were not stained. Micrographs were taken using a BZ-9000 microscope (Keyence) for measurement of myelin diameters. The G/T ratio (Figure S1A) was defined as the ratio of axon diameter to myelin diameter.

#### RT-PCR

RT-PCR was performed as described.^46^ Total RNA was isolated from DRGs using an RNeasy Mini kit (Qiagen, Hilden, Germany), according to the manufacturer’s instructions. Reverse transcription of RNA to cDNA was performed using a SuperScript Reverse Transcriptase III kit (Thermo Fisher Scientific) with random hexamer primers. Each cDNA sample was analyzed using quantitative PCR with the StepOnePlus Real-Time PCR System (Thermo Fisher Scientific). Gene mRNA levels were determined by RT-qPCR using TaqMan probes (Thermo Fisher Scientific) (Table S2). Samples were run in triplicate and relative fold-changes in mRNA levels were calculated using the 2^-ΔΔCt^ method.

#### RNA sequence analyses

Mouse DRG samples were also collected for RNA-seq. Total RNA was isolated using an RNeasy Mini kit (Qiagen), and RNA-seq was analyzed by Takara Bio, Inc. (Shiga, Japan). Libraries were prepared by following the SMART-Seq v4 Ultra Low Input RNA Kit (Clontech Laboratories, Moutain View, CA, USA) user manual. In brief, RNA samples were evaluated with the Agilent 4200 TapeStation (Santa Clara, CA, USA), and 1 μg samples with RNA Integrity Number (RIN) > 7.0 were used for library preparation. Specific sequences were added to both ends of the first stand DNA using the SMART (Switching Mechanism At 5’ End of RNA Template) method. Subsequently, PCR was performed using primers that recognize specific sequences to obtain double stranded cDNA following purification of PCR products by the magnetic bead method using AMPure XP (Beckman Coulter, Brea, CA, USA). The double stranded cDNA (0.2 ng) was amplified with 11 cycles of PCR. A Nextera XT DNA Library Preparation Kit and a Nextera XT Index kit v2 SetA/B/C/D (Illumina, San Diego, CA, USA) were used to produce sequence libraries suitable for Illumina sequencing. The quality of the prepared sequence libraries was measured using an Agilent 2100 BioAnalyzer. Finally, optimal cluster density was determined by quantitation of sequence libraries according to the Illumina NovaSeq 6000 sequencing System Guide v11. One hundred and fifty bp paired-end sequence reads were generated using an Illumina NovaSeq 6000 with NovaSeq 6000 S4 Reagent Kit/NovaSeq Xp 4-Lane Kit.

A Student’s t-test was performed to compare the diabetic WT group expression values to the other control samples with R (https://www.r-project.org/). FDR corrections for the P values were carried out with the ‘Benjamini & Hochberg’ method.^47^ Gene Set Enrichment Analysis (GSEA) was performed to uncover significant functional categories and pathways among the set of expressed genes between the diabetic WT group and the other control samples.^48^ In detail, gene synonyms were mapped by mouse ortholog to the Human Molecular Signatures Database (MSigDB, Ver.4.0.3). The expression fold changes between the upregulated and downregulated gene groups were then utilized for the pre-ranked function of the GSEA software.

#### Dissociated cell culture of DRG neurons

DRGs were excised and treated with 0.2% collagenase type III (Worthington Biochemical, Lakewood, NJ08701, USA) for 90 min, and then with 0.25% trypsin type III (Sigma-Aldrich) for 15 min at 37°C. The enzyme reaction was stopped by the addition of trypsin inhibitor (Sigma-Aldrich), and cells were collected by centrifugation using 30% Percoll (GE Healthcare, Boston, MA, USA). A total of 1×10^4^ neuronal cells were cultured using DMEM with 1% penicillin-streptomycin (WAKO) for 72 h, and the collected culture supernatants were divided into two samples. The first and second portions were supplemented with a TGF-β receptor inhibitor, SB431542, or dimethyl sulfoxide (solvent), respectively. MS1 (Mile Sven-1) endothelial cells were pre-treated overnight in DMEM with 5 μM SB431542; then, the medium was replaced and incubated for 4 h. The MS1 cells were subjected to RNA extraction and quantitative RT-PCR for expression of *Pmepa1*, a direct target gene of TGF-β signals, in order to examine the effect of the active form of TGF-β in the supernatants.

#### Western blotting

Sciatic nerves isolated from mice were washed with cold PBS and immediately frozen in liquid nitrogen. The frozen sciatic nerves were individually ground with a chilled micro-glass homogenizer, and pulverized samples were re-homogenized in 2xSDS sample buffer (125 mM Tris-HCl [pH 6.8], 4% SDS, 20% glycerol, 100 mM DTT). Proteins were separated by SDS-PAGE using 7.5% acrylamide gels and transferred to PVDF membranes. Membranes were blocked in 3% BSA in TBS-T for 1 h at room temperature and incubated with primary antibodies in 3% BSA in TBS-T overnight at 4°C. Anti-Smad2/3 antibody (1:500 dilution, R&D Systems), anti-phosphorylated Smad2 (pSmad S245/250/255) antibody (1:500 dilution, Cell Signaling Technology, Danvers, MA 01923, USA), anti-SAPK/JNK antibody (1:500 dilution, Cell Signaling Technology), and anti-phosphorylated SAPK/JNK (Y183/Y185:81E11) antibody (1:500 dilution, Cell Signaling Technology) were used as primary antibodies. After incubation with secondary antibodies for 3 h at room temperature, proteins were visualized using enhanced chemiluminescence (ECL™ Prime Western blotting system, Amersham, GE HealthCare) and detected by chemiluminescence “Light-CaptureII” with a CS Analyzer3.0 (ATTO, Tokyo, Japan). Densitometric analysis of the band intensities was performed using Fiji, ImageJ (NIH). For re-staining with different antibodies on the same blot, stripping of the antibodies from membranes was performed by incubation in stripping solution (100 mM 2-mercaptoethanol, 2% (w/v) SDS, 62.5 mM Tris-HCl, pH 6.7) for 30 min at 50°C after each analysis.

#### Anti-PDGFRβ antibody injection

Rat anti-mouse PDGFRβ monoclonal antibody (clone APB5)^25^ was dissolved in PBS at 1 mg/ml, and 30 μl of the reagent was injected intraperitoneally once on postnatal day 1. In the control group, only PBS was injected. On postnatal day 8, the mice were euthanized and their retinas were extracted for IHC studies.

#### Metabolome analysis

For metabolome analysis, mice were fasted for 12 h and sciatic nerves were extracted soon after euthanasia and kept at – 80°C. These samples were analyzed by Human Metabolome Technologies, Inc. (Tokyo, Japan) using a capillary electrophoresis time-of-flight mass spectrometry (CE-TOFMS) system. In brief, frozen sciatic nerve tissue was placed in a homogenization tube, along with zirconia beads. Next, 1200–1500 μl of 50% acetonitrile/Milli-Q water containing internal standards (H3304-1002, Human Metabolome Technologies, Inc. (HMT), Yamagata, Japan) was added to the tube, after which the tissue was completely homogenized at 3500 rpm, 4°C for 540 s using a bead shaker (Shake Master NEO, Bio Medical Science, Tokyo, Japan). The homogenate was then centrifuged at 2300 × g for 5 min at 4°C. Subsequently, 400 μl of the upper aqueous layer was centrifugally filtered through a Millipore 5-kDa cutoff filter (UltrafreeMC-PLHCC, HMT) at 9,100 × g, 4°C for 180 min to remove macromolecules. The filtrate was evaporated to dryness under vacuum and reconstituted in 50 μl of Milli-Q water for metabolome analysis at HMT. Metabolome analysis was conducted according to HMT’s Basic Scan package using CE-TOFMS, based on the methods.^49^^,^^50^

### Quantification and statistical analysis

Student’s *t*-test, one-way analysis of variance (ANOVA) with Tukey’s test or a two-way ANOVA with Bonferroni’s test were performed for multiple testing using GraphPad Prism (GraphPad Software, La Jolla, CA, USA). Values of *p* < 0.05 were considered to be statistically significant.

## References

[1] Pearson E.R. (2019). Type 2 diabetes: a multifaceted disease. Diabetologia.

[2] Galicia-Garcia U., Benito-Vicente A., Jebari S., Larrea-Sebal A., Siddiqi H., Uribe K.B., Ostolaza H., Martín C. (2020). Pathophysiology of Type 2 Diabetes Mellitus. Int. J. Mol. Sci..

[3] Feldman E.L., Callaghan B.C., Pop-Busui R., Zochodne D.W., Wright D.E., Bennett D.L., Bril V., Russell J.W., Viswanathan V. (2019). Diabetic neuropathy. Nat. Rev. Dis. Prim..

[4] Feldman E.L., Nave K.A., Jensen T.S., Bennett D.L.H. (2017). New Horizons in Diabetic Neuropathy: Mechanisms, Bioenergetics, and Pain. Neuron.

[5] Dewanjee S., Das S., Das A.K., Bhattacharjee N., Dihingia A., Dua T.K., Kalita J., Manna P. (2018). Molecular mechanism of diabetic neuropathy and its pharmacotherapeutic targets. Eur. J. Pharmacol..

[6] Eid S.A., Rumora A.E., Beirowski B., Bennett D.L., Hur J., Savelieff M.G., Feldman E.L. (2023). New perspectives in diabetic neuropathy. Neuron.

[7] Jafarzadeh F., Javanbakht A., Bakhtar N., Dalvand A., Shabani M., Mehrabinejad M.M. (2022). Association between diabetic retinopathy and polymorphisms of cytokine genes: a systematic review and meta-analysis. Int. Ophthalmol..

[8] Oza M.J., Laddha A.P., Gaikwad A.B., Mulay S.R., Kulkarni Y.A. (2021). Role of dietary modifications in the management of type 2 diabetic complications. Pharmacol. Res..

[9] Cole J.B., Florez J.C. (2020). Genetics of diabetes mellitus and diabetes complications. Nat. Rev. Nephrol..

[10] Forbes J.M., Cooper M.E. (2013). Mechanisms of diabetic complications. Physiol. Rev..

[11] Mikami T., Kitagawa H. (2013). Biosynthesis and function of chondroitin sulfate. Biochim. Biophys. Acta.

[12] Walimbe T., Panitch A. (2019). Proteoglycans in Biomedicine: Resurgence of an Underexploited Class of ECM Molecules. Front. Pharmacol..

[13] Igarashi M., Takeuchi K., Sugiyama S. (2018). Roles of CSGalNAcT1, a key enzyme in regulation of CS synthesis, in neuronal regeneration and plasticity. Neurochem. Int..

[14] Watanabe Y., Takeuchi K., Higa Onaga S., Sato M., Tsujita M., Abe M., Natsume R., Li M., Furuichi T., Saeki M. (2010). Chondroitin sulfate N-acetylgalactosaminyltransferase-1 is required for normal cartilage development. Biochem. J..

[15] Mizumoto S., Yamada S., Sugahara K. (2015). Molecular interactions between chondroitin-dermatan sulfate and growth factors/receptors/matrix proteins. Curr. Opin. Struct. Biol..

[16] Takeuchi K., Yoshioka N., Higa Onaga S., Watanabe Y., Miyata S., Wada Y., Kudo C., Okada M., Ohko K., Oda K. (2013). Chondroitin sulphate *N*-acetylgalactosaminyl-transferase-1 inhibits recovery from neural injury. Nat. Commun..

[17] Inada R., Miyamoto K., Tanaka N., Moriguchi K., Kadomatsu K., Takeuchi K., Igarashi M., Kusunoki S. (2021). Chondroitin sulfate *N*-acetylgalactosyltransferase-1 knockout shows milder phenotype in experimental autoimmune encephalomyelitis than in wild type. Glycobiology.

[18] Sango K., Mizukami H., Horie H., Yagihashi S. (2017). Impaired Axonal Regeneration in Diabetes. Perspective on the Underlying Mechanism from *In Vivo* and *In Vitro* Experimental Studies. Front. Endocrinol..

[19] Kolset S.O., Reinholt F.P., Jenssen T. (2012). Diabetic nephropathy and extracellular matrix. J. Histochem. Cytochem..

[20] Massagué J., Sheppard D. (2023). TGF-β signaling in health and disease. Cell.

[21] Benarroch E.E. (2011). CGRP: sensory neuropeptide with multiple neurologic implications. Neurology.

[22] Harrison S., Geppetti P. (2001). Substance P. Int. J. Biochem. Cell Biol..

[23] Takeshita Y., Sato R., Kanda T. (2020). Blood-Nerve Barrier (BNB) Pathology in Diabetic Peripheral Neuropathy and In Vitro Human BNB Model. Int. J. Mol. Sci..

[24] Stallcup W.B. (2018). The NG2 Proteoglycan in Pericyte Biology. Adv. Exp. Med. Biol..

[25] Ogura S., Kurata K., Hattori Y., Takase H., Ishiguro-Oonuma T., Hwang Y., Ahn S., Park I., Ikeda W., Kusuhara S. (2017). Sustained inflammation after pericyte depletion induces irreversible blood-retina barrier breakdown. JCI Insight.

[26] Kandasamy M., Anusuyadevi M., Aigner K.M., Unger M.S., Kniewallner K.M., de Sousa D.M.B., Altendorfer B., Mrowetz H., Bogdahn U., Aigner L. (2020). TGF-β Signaling: A Therapeutic Target to Reinstate Regenerative Plasticity in Vascular Dementia?. Aging Dis..

[27] Kamato D., Little P.J. (2020). Smad2 linker region phosphorylation is an autonomous cell signalling pathway: Implications for multiple disease pathologies. Biomed. Pharmacother..

[28] Pang L., Lian X., Liu H., Zhang Y., Li Q., Cai Y., Ma H., Yu X. (2020). Understanding Diabetic Neuropathy: Focus on Oxidative Stress. Oxid. Med. Cell. Longev..

[29] Holahan M.R. (2017). A Shift from a Pivotal to Supporting Role for the Growth-Associated Protein (GAP-43) in the Coordination of Axonal Structural and Functional Plasticity. Front. Cell. Neurosci..

[30] Kawasaki A., Okada M., Tamada A., Okuda S., Nozumi M., Ito Y., Kobayashi D., Yamasaki T., Yokoyama R., Shibata T. (2018). Growth Cone Phosphoproteomics Reveals that GAP-43 Phosphorylated by JNK Is a Marker of Axon Growth and Regeneration. iScience.

[31] Horton W.B., Barrett E.J. (2021). Microvascular Dysfunction in Diabetes Mellitus and Cardiometabolic Disease. Endocr. Rev..

[32] Cameron N.E., Eaton S.E., Cotter M.A., Tesfaye S. (2001). Vascular factors and metabolic interactions in the pathogenesis of diabetic neuropathy. Diabetologia.

[33] Nukada H. (2014). Ischemia and diabetic neuropathy. Handb. Clin. Neurol..

[34] Itoh S., Itoh F. (2018). TMEPAI family: involvement in regulation of multiple signalling pathways. J. Biochem..

[35] Wheeler S.E., Lee N.Y. (2017). Emerging Roles of Transforming Growth Factor β Signaling in Diabetic Retinopathy. J. Cell. Physiol..

[36] Wang L., Wang H.L., Liu T.T., Lan H.Y. (2021). TGF-Beta as a Master Regulator of Diabetic Nephropathy. Int. J. Mol. Sci..

[37] Rostam M.A., Kamato D., Piva T.J., Zheng W., Little P.J., Osman N. (2016). The role of specific Smad linker region phosphorylation in TGF-β mediated expression of glycosaminoglycan synthesizing enzymes in vascular smooth muscle. Cell. Signal..

[38] Kamato D., Burch M., Zhou Y., Mohamed R., Stow J.L., Osman N., Zheng W., Little P.J. (2019). Individual Smad2 linker region phosphorylation sites determine the expression of proteoglycan and glycosaminoglycan synthesizing genes. Cell. Signal..

[39] Gris P., Tighe A., Levin D., Sharma R., Brown A. (2007). Transcriptional regulation of scar gene expression in primary astrocytes. Glia.

[40] Prante C., Milting H., Kassner A., Farr M., Ambrosius M., Schön S., Seidler D.G., Banayosy A.E., Körfer R., Kuhn J. (2007). Transforming growth factor beta1-regulated xylosyltransferase I activity in human cardiac fibroblasts and its impact for myocardial remodeling. J. Biol. Chem..

[41] Abbott C.A., Chaturvedi N., Malik R.A., Salgami E., Yates A.P., Pemberton P.W., Boulton A.J.M. (2010). Explanations for the lower rates of diabetic neuropathy in Indian Asians versus Europeans. Diabetes Care.

[42] Izumikawa T., Koike T., Shiozawa S., Sugahara K., Tamura J.i., Kitagawa H. (2008). Identification of chondroitin sulfate glucuronyltransferase as chondroitin synthase-3 involved in chondroitin polymerization: chondroitin polymerization is achieved by multiple enzyme complexes consisting of chondroitin synthase family members. J. Biol. Chem..

[43] Quarta S., Baeumer B.E., Scherbakov N., Andratsch M., Rose-John S., Dechant G., Bandtlow C.E., Kress M. (2014). Peripheral nerve regeneration and NGF-dependent neurite outgrowth of adult sensory neurons converge on STAT3 phosphorylation downstream of neuropoietic cytokine receptor gp130. J. Neurosci..

[44] Uemura A., Ogawa M., Hirashima M., Fujiwara T., Koyama S., Takagi H., Honda Y., Wiegand S.J., Yancopoulos G.D., Nishikawa S.I. (2002). Recombinant angiopoietin-1 restores higher-order architecture of growing blood vessels in mice in the absence of mural cells. J. Clin. Invest..

[45] Shimizu H., Yamada M., Matsubara N., Takano H., Umeda Y., Kawase Y., Kitamoto T., Nishizawa M., Takahashi H. (2009). Creutzfeldt-Jakob disease with an M232R substitution: report of a patient showing slowly progressive disease with abundant plaque-like PrP deposits in the cerebellum. Neuropathology.

[46] Cho K., Ushiki T., Ishiguro H., Tamura S., Araki M., Suwabe T., Katagiri T., Watanabe M., Fujimoto Y., Ohashi R. (2021). Altered microbiota by a high-fat diet accelerates lethal myeloid hematopoiesis associated with systemic SOCS3 deficiency. iScience.

[47] Benjamini Y., Hochberg Y. (1995). Controlling the False Discovery Rate: a Practical and Powerful Approach to Multiple Testing. J. Roy. Stat. Soc. B.

[48] Subramanian A., Tamayo P., Mootha V.K., Mukherjee S., Ebert B.L., Gillette M.A., Paulovich A., Pomeroy S.L., Golub T.R., Lander E.S., Mesirov J.P. (2005). Gene set enrichment analysis: A knowledge-based approach for interpreting genome-wide expression profiles. Proc. Natl. Acad. Sci. USA.

[49] Ohashi Y., Hirayama A., Ishikawa T., Nakamura S., Shimizu K., Ueno Y., Tomita M., Soga T. (2008). Depiction of metabolome changes in histidine-starved Escherichia coli by CE-TOFMS. Mol. Biosyst..

[50] Ooga T., Sato H., Nagashima A., Sasaki K., Tomita M., Soga T., Ohashi Y. (2011). Metabolomic anatomy of an animal model revealing homeostatic imbalances in dyslipidaemia. Mol. Biosyst..

