# Supplementary material for: Reduced chondroitin sulfate content prevents diabetic neuropathy through transforming growth factor-β signaling suppression
Source: iScience. 2024 Mar 18;27(4):109528. doi: 10.1016/j.isci.2024.109528 (PMC11002665; doi:10.1016/j.isci.2024.109528)
Supplement: Document S1. Figures S1–S4 and Tables S1 and S2 [file mmc1.pdf]

iScience, Volume *27*

Supplemental information

Reduced chondroitin sulfate content prevents diabetic neuropathy through transforming growth factor-β signaling suppression

Hajime Ishiguro, Takashi Ushiki, Atsuko Honda, Yasuhiro Yoshimatsu, Riuko Ohashi, Shujiro Okuda, Asami Kawasaki, Kaori Cho, Suguru Tamura, Tatsuya Suwabe, Takayuki Katagiri, Yiwei Ling, Atsuhiko Iijima, Tadahisa Mikami, Hiroshi Kitagawa, Akiyoshi Uemura, Kazunori Sango, Masayoshi Masuko, Michihiro Igarashi, and Hirohito Sone

Supplemental information

Reduced chondroitin sulfate content prevents diabetic neuropathy through TGF-β signaling suppression

Hajime Ishiguro, Takashi Ushiki, Atsuko Honda, Yasuhiro Yoshimatsu, Riuko Ohashi, Shujiro Okuda, Asami Kawasaki, Kaori Cho, Suguru Tamura, Tatsuya Suwabe, Takayuki Katagiri, Yiwei Ling, Atsuhiko Iijima, Tadahisa Mikami, Hiroshi Kitagawa, Akiyoshi Uemura, Kazunori Sango, Masayoshi Masuko, Michihiro Igarashi, and Hirohito Sone

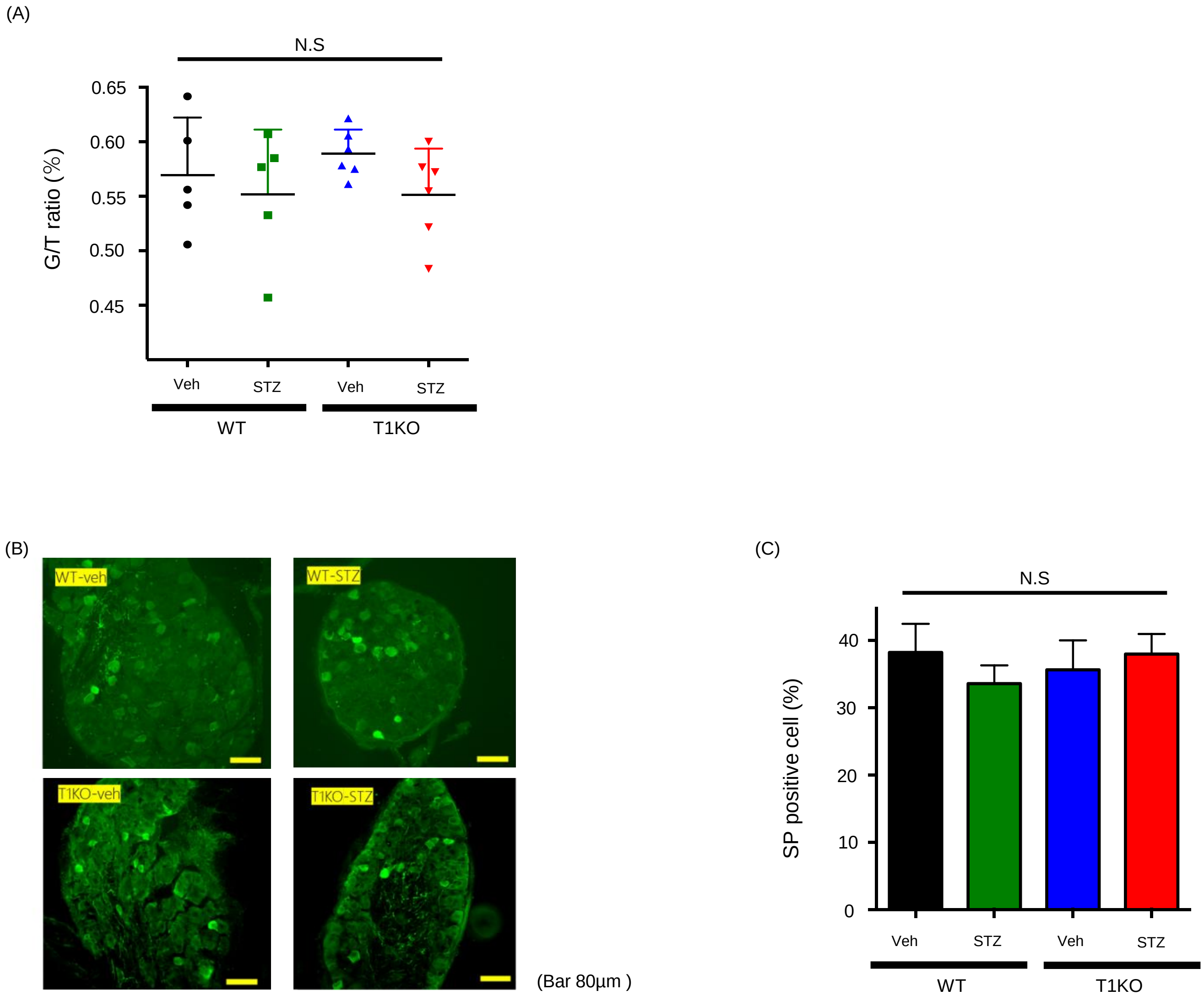

Figure S1. DN parameters that were not significantly different between the diabetic WT and T1KO groups, related to Figure 2.

(A) The G/T ratio (axon diameter / fiber diameter) of the sciatic nerve. The average ratio of 300 blue-stained fibers per mouse at 3 weeks. Mean ± SD is shown, n = 5 mice in non-diabetic WT group and diabetic WT group, n = 6 mice in non-diabetic T1KO group and diabetic T1KO group. **(B)** Representative images of substance P staining in the DRG. Scale bar = 80 μm. **(C)** The ratio of substance P-positive neurons in the DRG. Neurons were quantitated from five DRGs per mouse. Mean ± SD is shown, n = 3 mice per group.

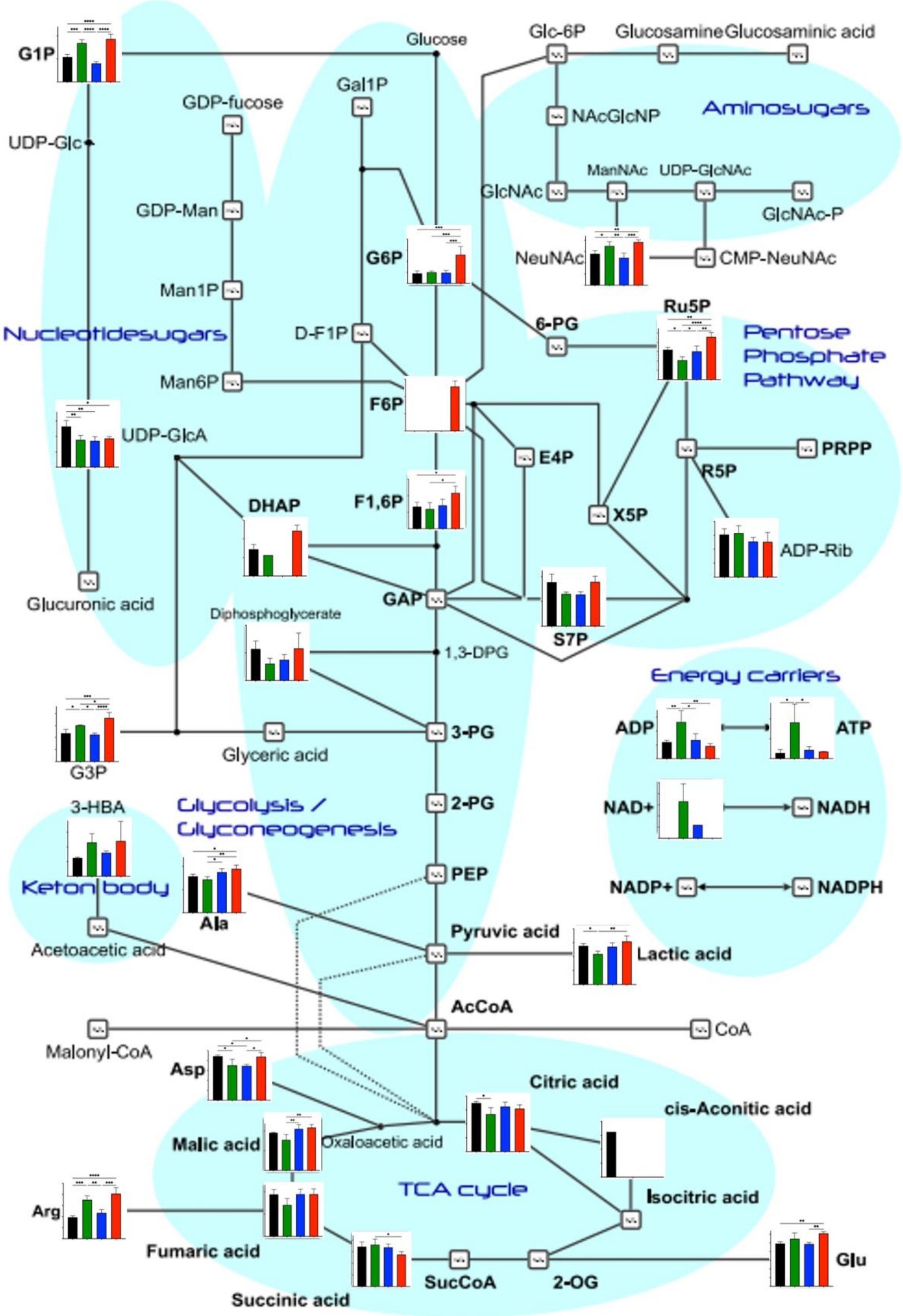

Figure S2. **Metabolome analysis of sciatic nerves in each group, shown as a metabolic map focused on glycolysis and the TCA cycle, related to Figure 1 and Figure 2**. Data in the bar graphs is as follows: *Black*, WT-vehicle; *Green*, WT-STZ; *Blue*, T1KO-vehicle; *Red*, T1KO-STZ. Mean ± SD is shown with *p < 0.05, **p < 0.01, ***p < 0.001, and ****p < 0.0001 for comparison, one-way ANOVA with Tukey's multiple comparisons test, n = 3 in each group.

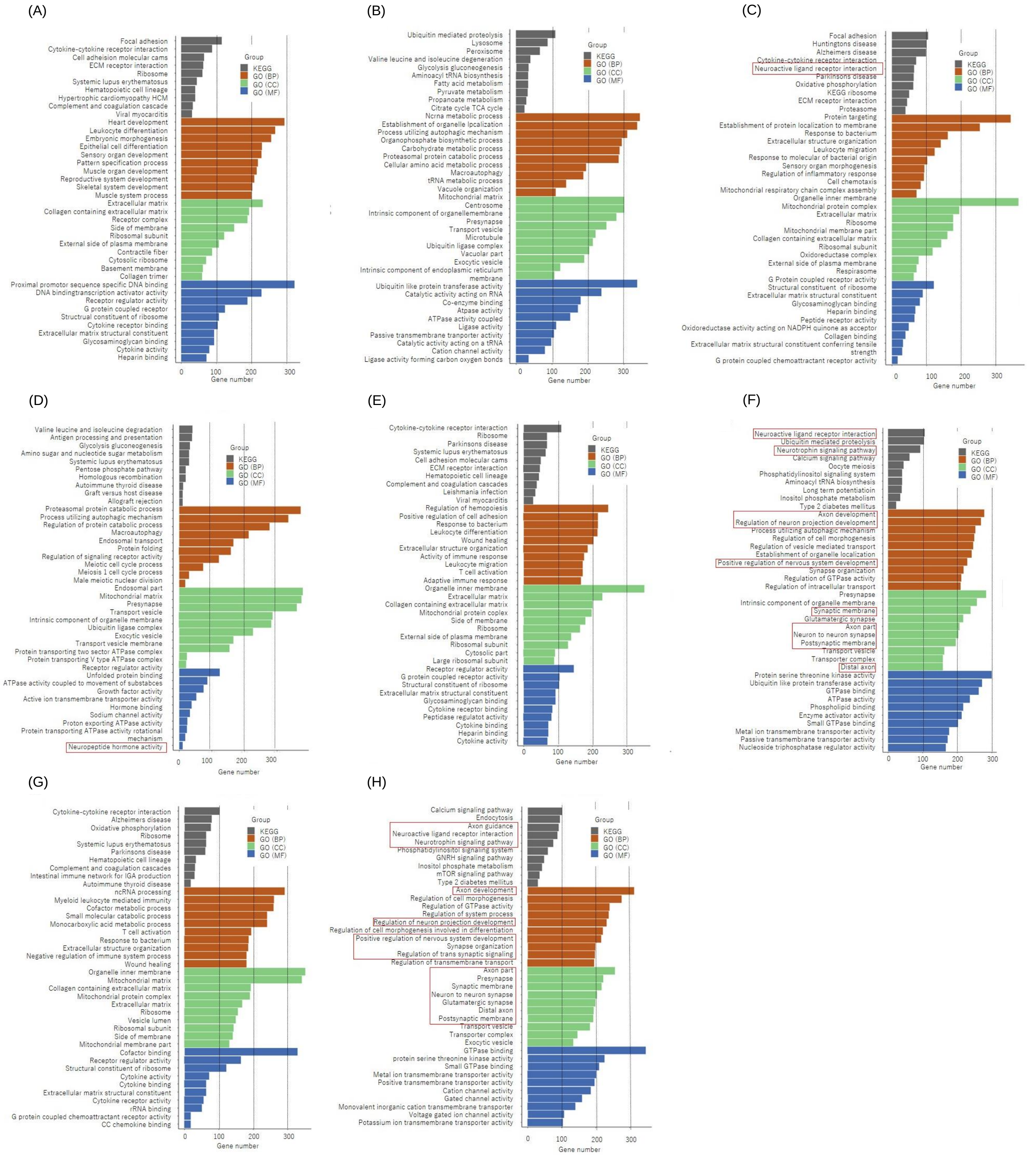

Figure S3. Top ten categories, defined as GO or KEGG terms, with significantly altered gene expression among the groups, related to Figure 3 and Figure 5. **(A)** Upregulation in WT-STZ vs WT-vehicle, **(B)** downregulation in WT-STZ vs WT-vehicle, **(C)** upregulation in T1KO-vehicle vs WT-vehicle, **(D)** downregulation in T1KO-vehicle vs WT-vehicle, **(E)** upregulation in T1KO-STZ vs T1KO-vehicle, **(F)** downregulation in T1KO-STZ vs T1KO-vehicle, **(G)** upregulation in T1KO-STZ vs WT-STZ, and **(H)** downregulation in T1KO-STZ vs WT-STZ. Red boxes indicated genes associated with nerves.

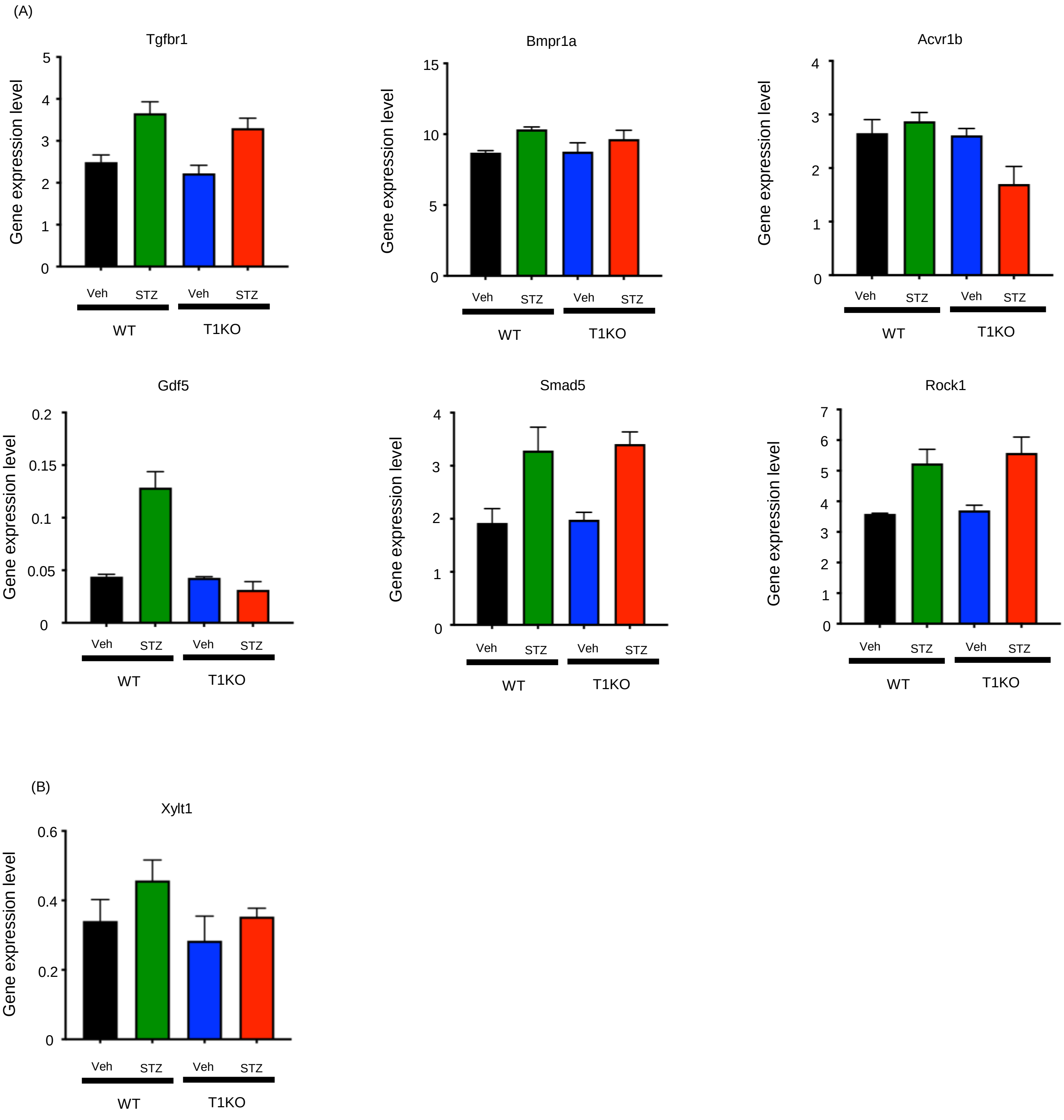

Figure S4. Diabetic T1KO mice tended to be weaker in TGF-β-related signaling than diabetic WT, related to Figure 5. (A) Gene expressions related to TGF-β and TGF-β-related receptors extracted from RNA-sequencing: *Tgfbr1, Bmpr1a, Acvr1b, Gdf1, Smad5*, and *Rock1.* **(B)** Gene expression of *Xylt1*, the initiating enzyme of CS synthesis. n = 3 in each group.

Table S1. mRNAs of TGF-β superfamily receptor-related signaling molecules were not elevated in diabetic T1KO mice, related to Figure 5.

Gene expression extracted from RNA-Seq. Transforming growth factor-β receptor 1 (*Tgfbr1*), bone morphogenetic protein receptor type 1A *(Bmpr1a),* activin receptor type 1B (*Acvr1b),* growth differentiation factor 1 (*Gdf1), Smad5,* and rho associated coiled-coil containing protein kinase 1 (*Rock1)* are receptors of TGF-β superfamily and its related diffusible factors; xylosyltransferase 1 (*Xylt1)* is an initiation enzyme of CS synthesis. n = 3 per group.

gene name	WT-vehicle			WT-STZ			T1-vehicle			T1-STZ			WT-STZ/Others		
													logFC	P-value	Q-value
Tgfbr1	2.56463	2.61519	2.29507	3.65246	3.38395	3.93445	2.43341	2.18716	2.04811	3.52972	3.32517	3.05519	0.45238	0.004	0.24774
Bmpr1a	8.55257	8.83333	8.70837	10.22136	10.53528	10.26649	9.30946	8.90415	8.08512	10.33598	9.50703	9.11422	0.19416	0.00025	0.09861
Acvr1b	2.41787	2.61509	2.92036	3.01294	2.91234	2.69341	2.51965	2.55188	2.75782	1.33179	1.82015	1.95398	0.30779	0.01804	0.4409
Gdf5	0.0447	0.0457	0.04128	0.14372	0.11343	0.12882	0.04391	0.04168	0.0429	0.02846	0.02539	0.04022	1.70873	0.00611	0.29048
Smad5	2.02031	2.12657	1.61527	3.04647	3.0164	3.79487	1.91771	1.88527	2.14102	3.66664	3.30608	3.24609	0.43172	0.05306	0.62138
Rock1	3.61291	3.57387	3.58274	4.94541	5.00545	5.77031	3.52322	3.85323	3.739	5.13825	6.14989	5.45227	0.28812	0.05637	0.63342
Xylt1	0.33687	0.2813	0.40433	0.20406	0.34022	0.3071	0.46775	0.39373	0.51036	0.3427	0.3362	0.38093	0.48835	0.0363	0.55138

Table S2. Probes used for qPCR, related to STAR METHOD.

Primers	Assay ID
Caspase 3	Mm01195085_m1
Caspase 9	Mm00516563_m1
B cell lymphoma-2 (bcl-2)	Mm00477631_m1
Tumor Necrosis Factor α (TNF-α)	Mm00443258_m1
Matrix metalloproteinase 9 (MMP-9)	Mm00442991_m1
Interleukin-6 (IL-6)	Mm00446190_m1
heme oxygenase 1 (Ho-1)	Mm00516005_m1
NADPH oxidase (NOX)	Mm00549170_m1
Interleukin-18 (IL-18)	Mm00434226_m1
Interleukin -1β (IL-1β)	Mm00434228_m1
Caspase 1	Mm00438023_m1
GAPDH	Mm99999915_g1
